# High-Precision Registration of Point Clouds Based on Sphere Feature Constraints

**DOI:** 10.3390/s17010072

**Published:** 2016-12-30

**Authors:** Junhui Huang, Zhao Wang, Jianmin Gao, Youping Huang, David Peter Towers

**Affiliations:** 1 Key Laboratory of Education Ministry for Modern Design and Rotor-Bearing System, School of Mechanical Engineering, Xi’an Jiaotong University, Xi’an 710049, China; huangyouping@stu.xjtu.edu.cn; 2 State Key Laboratory for Manufacturing Systems Engineering, Xi’an Jiaotong University, Xi’an 710049, China; gjm@mail.xjtu.edu.cn; 3 School of Engineering, University of Warwick, Coventry CV4 7AL, UK; D.Towers@warwick.ac.uk

**Keywords:** 3D measurement, point cloud, registration, virtual overlapping areas, feature constraint

## Abstract

Point cloud registration is a key process in multi-view 3D measurements. Its precision affects the measurement precision directly. However, in the case of the point clouds with non-overlapping areas or curvature invariant surface, it is difficult to achieve a high precision. A high precision registration method based on sphere feature constraint is presented to overcome the difficulty in the paper. Some known sphere features with constraints are used to construct virtual overlapping areas. The virtual overlapping areas provide more accurate corresponding point pairs and reduce the influence of noise. Then the transformation parameters between the registered point clouds are solved by an optimization method with weight function. In that case, the impact of large noise in point clouds can be reduced and a high precision registration is achieved. Simulation and experiments validate the proposed method.

## 1. Introduction

Three-dimensional (3D) profile measurement has been widely applied in reverse engineering, heritage preserving, 3D body scanning, and other areas [1,2,3,4]. Multi-view measurement is used often for a large size object, and 3D point clouds of each view are registered into a complete point cloud. Therefore, a registration of 3D point clouds is a key technique to achieve high precision.

Point clouds registration is that the point cloud of each view is transformed into a same common coordinate system. There are various approaches [5,6,7,8,9,10,11,12,13,14,15,16,17,18,19,20,21] to register the multi-view 3D point clouds. Generally, the current registration methods can be classified into two categories. One is reference-based registration—such as marked-points method [5,6], global framework method [7,8], sensors tracking method [9,10], and so on—where feature targets or sensors are used as the reference to align the point clouds together. Generally, those methods do not need overlapping areas in the registered point clouds, but the registration precision is limited by the reference’s precision, especially for a large size object. Thus, they are usually called rough registration. The other is free registration—such as Iterative Closest Point (ICP) algorithm [11], 3D Normal Distributions Transform based method [12], Gaussian Mixture Models based method [13] and so on—in which the point clouds are aligned together by their own features from the measurement points. The most typical method of them is the ICP algorithm [11] or its improved algorithm [14,15]. Usually, those methods in that category can be achieved higher precision, which are called as fine registration. Their precision is dependent greatly on the accuracy of corresponding point pairs (or feature pairs) from the overlapping areas [11,14,15]. So the overlapping amount and accuracy of corresponding point pairs (feature pairs) affects the registration precision directly. However, for some point clouds with curvature invariance or little change, such as planes and spheres, it fails to locate the accurate corresponding point pairs (or feature pairs) and returns an incorrect result. Consequently, the free registration methods need not only overlapping areas but also obvious changes in surface curvature.

A high precision registration method based on sphere feature constraints is proposed to address above problems. Practically, sphere target-based method is a common and useful method adopted in registrations of point clouds [16,17,18,19], where the point clouds are registered by alignment of the sphere centers, and that method is fast and does not need overlapping areas either. However, it is an inaccurate registration because measurement errors cause errors in the fitting sphere centers, which is called rough registration and sphere center registration (SCR). Even use of the sphere model to register, the precision is still not improved much because of errors in sphere fitting [19]. So the sphere targets are not only used for rough registration, but also employed to construct overlapping areas and provide constraints for fine registration in our proposed method. It combines the advantages of the above two categories of methods. That is, it does not need overlapping areas or variant curvature in the measurement point clouds by constructing virtual overlapping areas, and it could achieve high precision with the ICP algorithm even for large size object. In our previous study, the thought and procedure have been introduced [20]. However, it did not analyze the principle or essence of that method, and it lacks of analysis of the factors affecting the performance of that method. This paper will clearly reveal its principle and discuss those factors in detail; and in solving the transformation parameters, a weight function is added in the cost function, which will reduce the impact of large noise and improve the registration stability.

The rest of this paper is organized as below. In Section 2, the principle of the proposed method is introduced. Section 3 describes its algorithm processes. In Section 4, simulation and experiments are presented to validate the method. And conclusions are drawn in Section 5.

## 2. Principle of Registration

To achieve high precision, the ICP algorithm is adopted, but it needs overlapping areas in the registered point clouds. So some sphere targets with specific features are introduced and fixed rigidly with the measured object. Those specific features are used to construct entire surfaces with the measurement points of parts of the targets. The constructed entire surfaces will have overlapping areas with the measurement point clouds. Actually, those overlapping areas do not exit, so they are named virtual overlapping areas. The constraints in the feature targets are used to reduce errors in the entire surface constructing. 

Then, the corresponding points to the measurement points for the ICP algorithm are obtained from the virtual overlapping areas instead of other actual measurement point clouds. It could avoid errors in the measurement points and provide more accurate corresponding point pairs because of the constructed surface’s ideality and continuity. 

Finally, a weight function is added in the solving the transformation matrices, which reduces the impact of large noise and improves the registration stability. Thus, it could calculate the transformation matrices R (rotation matrix) and T (translation vector) between different point clouds more accurately. The following will describe it in detail.

### 2.1. Constructing Virtual Overlapping Areas

Firstly, multiple sphere feature targets with different distances between each other are employed to construct the virtual overlapping areas. The essence of multi-targets is to use the distance difference between each other to distinguish one target from another and to determine their orientations relative to the same target. Those feature targets have symmetry, which is easy to construct the entire surface by parts of the measurement points of targets.

**Figure 1:**
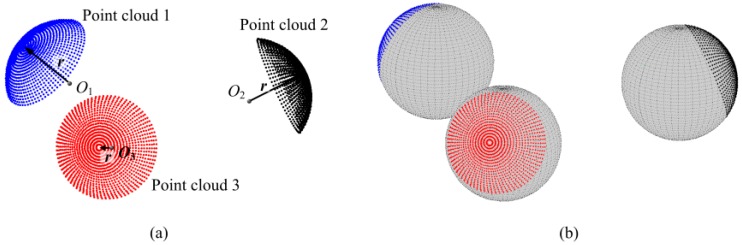
Construction of a sphere surface, (**a**) three point clouds measured from different perspectives of the same sphere target; (**b**) three sphere surfaces constructed by the measurement data of a part of a target.

The sphere center is calculated by a fitting method with the measurement points of any part of a sphere, as shown in Figure 1a, and the radius is measured in advance for a constraint to improve the constructing precision. Then the entire sphere surface can be constructed by its center and radius, as shown in Figure 1b. The spherical equation is set as:

(1) $${\left(x_{si}-x_{0}\right)}^{2}+{\left(y_{si}-y_{0}\right)}^{2}+{\left(z_{si}-z_{0}\right)}^{2}=r^{2}$$

where, ($x_{si},y_{si},z_{si}$) are the coordinates of a point in a sphere surface; $x_{0}$, $y_{0}$, and $z_{0}$ are the coordinates of sphere center; r is its radius. The radius of sphere target is measured in advance and set as a known parameter, and the $x_{0}$, $y_{0}$, and $z_{0}$ can be solved by minimizing the following function:

(2) $$\left\{ \begin{array}{l} f(x_{0},y_{0},z_{0})=\sum_{i=1}^{n}{\left[{\left(x_{si}-x_{0}\right)}^{2}+{\left(y_{si}-y_{0}\right)}^{2}+{\left(z_{si}-z_{0}\right)}^{2}-r^{2}\right]}^{2},\\ r=\mathrm{constant}. \end{array}\right.$$

where n is the number of fitting points. Minimizing Equation (2) is a nonlinear problem and it is solved with Levenberg-Marquardt algorithm [21]. Then an entire sphere surface is constructed with any pair of the sphere centers and radii, as shown in Figure 1b.

By constructing an entire feature surface, the constructed surface has overlapping areas with other measured point clouds. Due to the noise in the registered points, the fitting radius usually deviates from the actual value. Thus, the radius is constrained to a constant to avoid the fitting error.

### 2.2. Selection of Accurate Corresponding Point Pairs

As mentioned above, finding the correct corresponding point pairs is very crucial for registration with high precision. After the alignment of sphere centers, the constructed sphere surface is fitted by all the point clouds from different perspectives of the same target, which makes the constructed surface more reliable. Then each point $q_{i}$ in the registered point clouds selects its corresponding point ${\bar{q}}_{i}$ from the constructed surface instead of from other registered point clouds, even they have overlapping areas. That is, the *i*-th corresponding point of $q_{i}$ is obtained by Equation (3):

(3) $${\bar{q}}_{i}=r\cdot \frac{q_{i}-c_{0}}{\left\|q_{i}-c_{0}\right\|}+c_{0}$$

where, $c_{0}$ represents the sphere center and $c_{0}=(x_{0},y_{0},z_{0})$; $\left||q_{i}-c_{0}|\right|$ denotes the norm of vector ($q_{i}-c_{0}$), that is, $\left\|q_{i}-c_{0}\right\|=\sqrt{{\left(x_{i}-x_{0}\right)}^{2}+{\left(y_{i}-y_{0}\right)}^{2}+{\left(z_{i}-z_{0}\right)}^{2}}$ and ($x_{i},y_{i},z_{i}$) is the coordinates of $q_{i}$.

The constructed surface, providing overlapping areas, is error free and continuous, and is used as the reference for registered point clouds. In that case, the errors of corresponding points are avoided by selecting them from the ideal virtual overlapping areas. Accordingly, the impact of noise in measurement points is reduced and the wrong corresponding point pairs are avoided.

### 2.3. Solution of the Transformation Matrices **R** and **T**

The essence of the proposed registration method is that the measurement point clouds of the spheres are forcibly aligned to a united constructed sphere surface. With those corresponding point pairs ($q_{i}$ and ${\bar{q}}_{i}$), the rotation matrix R and translation vector T can be solved by a unit quaternions method [22], and the cost function is:

(4) $$f(R,T)=\frac{1}{N}\sum_{i=1}^{N}\left\|{\bar{q}}_{i}-(R\cdot q_{i}+T)\right\|$$

**Figure 2:**
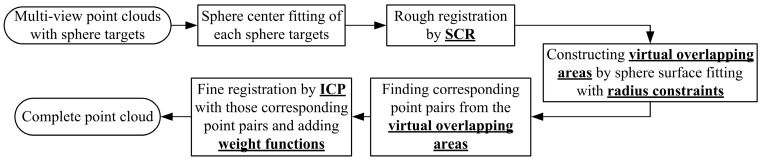
Workflow diagram of the FCR method.

Alternatively, a better option for the solution can be adopted by minimizing the following equation:

(5) $$f(R,T)=\frac{1}{N}\left\{\sum_{j=1}^{m}\left[\rho_{j}\cdot \sum_{i=1}^{n_{j}}{\left\|{\bar{q}}_{i}-(R\cdot q_{i}+T)\right\|}^{2}\right]\right\}$$

where, $q_{i}$ and ${\bar{q}}_{i}$ denote the vectors representing the *i*-th registered point in the registered point cloud and its corresponding point from the virtual overlapping areas, respectively; $n_{j}$ is the number of registration points in each point cloud; m is the number of targets; N is the total number of all points for registration; ||⋅|| denotes the Euclidean norm; $\rho_{j}$ denotes the weight of the *j*-th point cloud, its value is among [0, 1] and mainly depends on the measurement precision of the registered point clouds, that is,

(6) $$\rho_{j}=u_{j}^{-2}/\sum_{i=1}^{m}u_{i}^{-2}$$

where, $u_{j}$ is the bias between the fitting radius of a target with *j*-th point cloud and its actual radius. The weight $\rho_{j}$ is mainly used to improve the stability of registration, and the impact of some large errors in registered point clouds can be reduced by a small weight. The workflow of the proposed method is shown in Figure 2.

In summary, a high-precision registration method based on feature constraints (FCR) uses some specific features to construct an entire surface of the feature target and provide virtual overlapping areas. The feature constraint reduces the influence of noise and avoids the solution falling into a local optimum. By selecting corresponding points from the virtual overlapping areas, it could find more accurate corresponding point pairs to improve the registration precision. The solution of transformation parameters with weight function reduces the impact of large noise and improves the registration stability.

## 3. Algorithm Procedure

**Figure 3:**
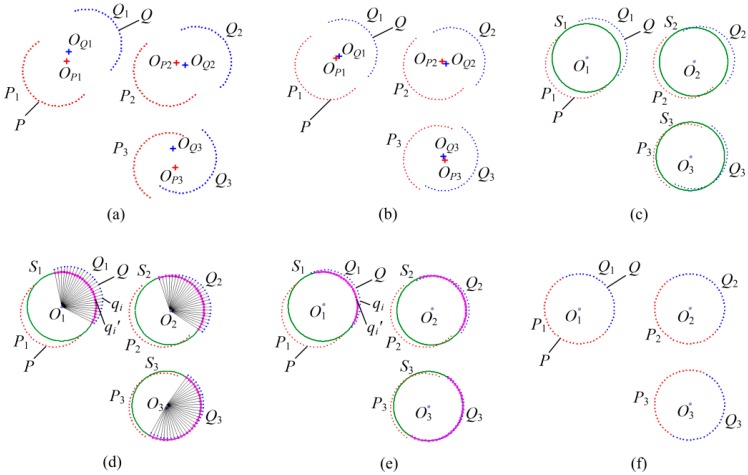
Two-dimensional schematic of the registration procedure of FCR: (**a**) identification of two point clouds to be registered; (**b**) rough registration by fitted sphere centers; (**c**) feature sphere fitting; (**d**) calculation of corresponding points; (**e**) solution of the rotation and translation matrices and (**f**) result of the registration.

Registration of two point clouds is introduced for an example. The procedure has been described in Reference [20], and a two-dimensional schematic of the registration procedure of FCR is shown in Figure 3, in which the procedure is described in more detail and the red and blue points represent different point clouds ***P*** and ***Q*** respectively. The registration procedure is illustrated in the following steps:

(1) Identification of the sphere point clouds. Use Hough transform to automatically detect the sphere point clouds from measurement point clouds [23], such as the sphere point clouds ***P*** and ***Q*** as shown in Figure 3a. ***P*1**, ***P*2**, ***P*3**, in ***P*** and ***Q*1**, ***Q*2**, ***Q*3**, in ***Q*** represent measurement point set from three different sphere targets, respectively. The radius r of sphere targets is measured by Coordinate Measuring Machine (CMM) in advance. For each sphere point cloud, the coordinates of sphere center are fitted by Equation (2). The distances between those centers are calculated with the solved center coordinates, and the distance differences are applied to distinguishing the targets, such as (*D_P_*_1*P*3_ ≈ *D_Q_*_1*Q*3_) > (*D_P_*_2*P*3_ ≈ *D_Q_*_2*Q*3_) > (*D_P_*_1*P*2_ ≈ *D_Q_*_1*Q*2_), shown in Figure 3a. That means ***P*_1_** and ***Q*_1_** from a same sphere target, ***P*_2_** and ***Q*_2_** from a same sphere target and ***P*_3_** and ***Q*_3_** from a same sphere target.
(2) Rough registration by sphere centers. The corresponding point clouds from the same target are registered by aligning the fitted sphere centers together (SCR) [16,17,18,19], as shown in Figure 3b. Rough registration provides the initial value of the transformation parameters.
(3) Construction of sphere surface. The coordinates of sphere center ***O*_1_~*O*_3_** are obtained by fitting the point clouds of the same target to a common sphere surface by Equation (2) respectively, while the radius r is set to a known value. The fitting residual is obtained by following Equation (7) and saved as *Err_Fit*.
  (7) $$Err\_Fit=\sum_{i=1}^{N}\left(\left|\left\|p_{i}-c_{0}\right\|-r\right|+\left|\left\|q_{i}-c_{0}\right\|-r\right|\right)$$
  where, ***p*_i_**, ***q*_i_** denote the vectors in point clouds ***P*** and ***Q***, respectively; ***c***_0_ is the fitting sphere center; |·| denotes calculating the absolute value.

With the fitted coordinates of sphere center and the known radius, continuous sphere surfaces are constructed. Each constructed sphere surface *S_j_* has overlapping areas with all the corresponding measurement point clouds. The constructed surfaces are represented in green as shown in Figure 3c.

(4) Selection of corresponding point pairs. Fix one point cloud, such as ***P***. Each point ***q*_i_** in ***Q*** is connected to the corresponding fitting sphere center ***O_j_*** (*j* = 1, 2, and 3), respectively. The connecting line intersects the sphere surface *S_j_* (*j* = 1, 2 and 3) at ***q_i_*'**, which is regarded as the corresponding point of ***q_i_***, as shown in Figure 3d. The coordinates of the intersection point ***q_i_*'** are calculated by Equation (3).
(5) Solution of the rotation matrix ***R*** and translation vector ***T***. With corresponding point pairs obtained in (4), a rotation matrix ***R*** and a translation vector ***T*** are solved by minimizing the Equation (5) (Improved-ICP). Then the point cloud ***Q*** is aligned to the constructed sphere surface with the solution of ***R*** and ***T***, as shown in Figure 3e. In fact, the fixed point cloud ***P*** can also be registered to the constructed surface simultaneously to accelerate the registration speed.
(6) Determination of iteration termination. Re-calculate the new fitting residuals (saved as *Err_Fit_New*) by Equation (7) with the point cloud ***P*** and the new point cloud ***Q*** obtained in (5), and calculate the variation of the fitting residuals *Vari_Fit* = |*Err_Fit_New* − *Err_Fit*|. Then determine whether the variation of fitting residuals or the solving residuals of Equation (5) in Step (5) is less than the setting value, or the number of iteration is larger than a setting number. When those happen, the iteration is terminated; otherwise return to Step (3). The final registration result is shown in Figure 3f.

## 4. Experiments and Analysis

### 4.1. Impact Analysis of Main Factors by Simulation

**Figure 4:**
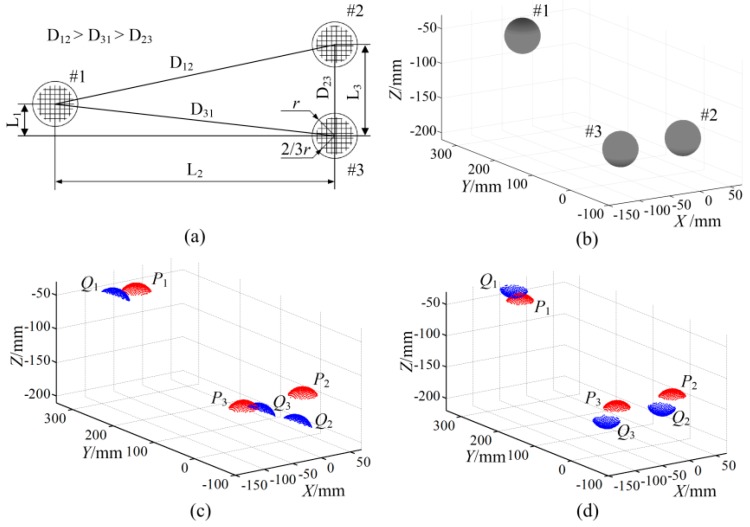
Simulation point clouds of sphere targets, (**a**) the relative positions of the sphere targets; (**b**) simulation sphere surface; (**c**) extracted point clouds with overlapping data; and (**d**) extracted point clouds with non-overlapping data.

To evaluate the proposed FCR method, three sphere surfaces are generated by simulation as shown in Figure 4a,b. Two simulations with and without overlapping data are carried out. The first two sphere point clouds (represented as ***P*** and ***Q***) are randomly extracted at equal intervals from the same upper spherical surfaces respectively: one is indicated by red (***P*_1_**~***P*_3_**) and the other is indicated by blue (***Q*_1_**~***Q*_3_**). The other two point clouds are extracted from the upper and lower spherical surfaces respectively, which have non-overlapping data.

Then a random Gaussian noise is added along the radius direction in those extraction points. It should be noted that those extraction points in the two point clouds are not coincident because their initial extraction positions are random and different, which is designed to simulate the actual measuring situation as much as possible. Then one of the point clouds is transformed to an arbitrary pose by rotation and translation. Figure 4c shows the transformed and fixed-point clouds with overlapping data, and Figure 4d shows the point clouds with non-overlapping data, where the transformed point cloud is indicated in blue and the fixed point cloud is indicated in red.

Generally, the target size, density of registration points, noise in registration points, and overlapping ratio may cause radius bias and position errors in sphere centers. So the registration precision is mainly affected by those factors. Simulations are conducted by varying the target size, density, noise level, and its anisotropy respectively, in which simulations are repeated 200 times with randomly re-generating simulation points. The mean value of the biases between the registered points and their theoretical positions is calculated for the registration result each time, and a mean value of those mean values is calculated for the final registration error, as following Equation (8):

(8) $$Err_{Mean}=\frac{1}{N_{times}}\cdot \sum \left[\frac{1}{N}\sum_{i=1}^{N}\left\|q_{i}^{'}-(R\cdot q_{i}+T)\right\|\right]$$

where, ***q*_i_** and ***q*_i_’** are the vectors representing the *i*-th registered point and its theoretical position respectively; ***R*** and ***T*** are the solution of the rotation matrix and translation vector respectively; *N* is the number of simulation points and *N_times_* is the simulation times. Then the final registration errors by Equation (8) are changed with only one variable in fixing other factors, which are shown in the following figures.

According to a common fringe projection measuring probe [24,25], the fixing values are set as: sphere radius *r* = 25.4 mm, point spacing (density) is 2 mm × 2 mm, mean value of the noise *u* = 0 and standard deviation of noise *σ* = 20 μm; the dimension of the point clouds is set to two-thirds of a hemisphere according to the field of view; the distances between the sphere targets are set to *L*_1_ = 36 mm, *L*_2_ = 315 mm, and *L*_3_ = 103 mm, as shown in Figure 4a.

#### 4.1.1. Impact of the Target Size

**Figure 5:**
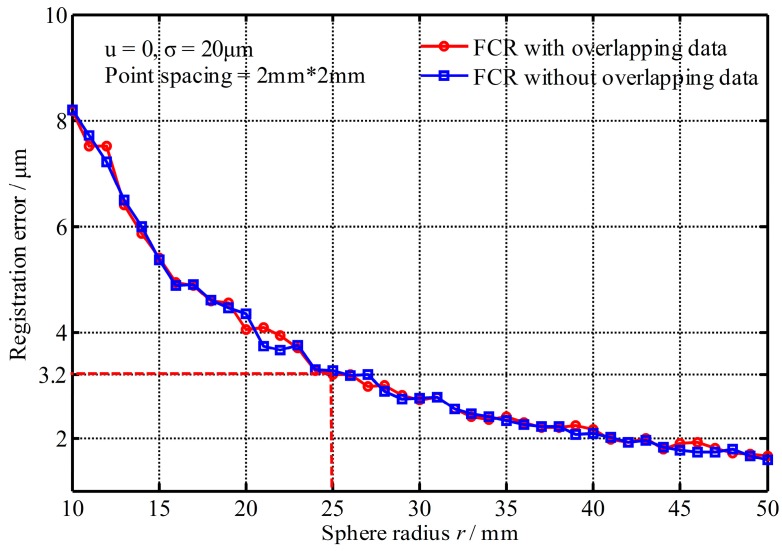
Registration errors changed with the sphere radius.

Figure 5 shows the registration errors changed with the sphere radius, where the registration errors decrease with the increase of target radius because more points improve the position accuracy of fitting sphere centers. However, when the radius is greater than 25 mm, the registration precision has a little improvement. Accordingly, the radius of sphere target set to *r* = 25.4 mm (1 inch) is adequate for registration in practice, where a mean error of 3.2 μm is achieved under the setting condition. From Figure 5, it also shows that the registration errors with non-overlapping data are similar to the results with overlapping data.

#### 4.1.2. Impact of the Point Density

**Figure 6:**
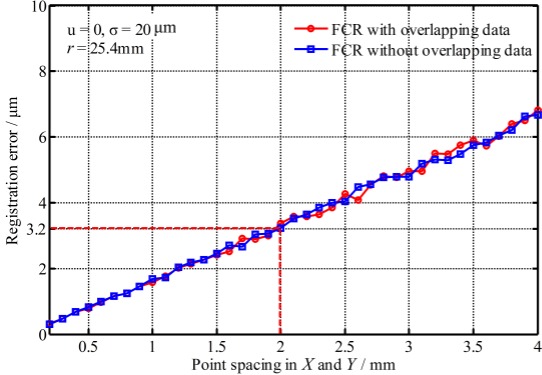
Registration errors changed with the point spacing.

Figure 6 shows the registration errors changed with the point density. It shows that the registration errors increase with the decrease of density and the registration errors with non-overlapping data are also similar to the results with overlapping data. Smaller point spacing could improve the registration precision, but the cost of measurement and data processing would increase greatly. Thus point spacing set to about 2 mm × 2 mm is acceptable under the setting condition, where a mean error of 3.2 μm is achieved.

#### 4.1.3. Impact of the Noise

The impact of noise is divided into two aspects, that is, the standard deviation of noise and the mean error, as below:

(1) Impact of the standard deviation of noise

**Figure 7:**
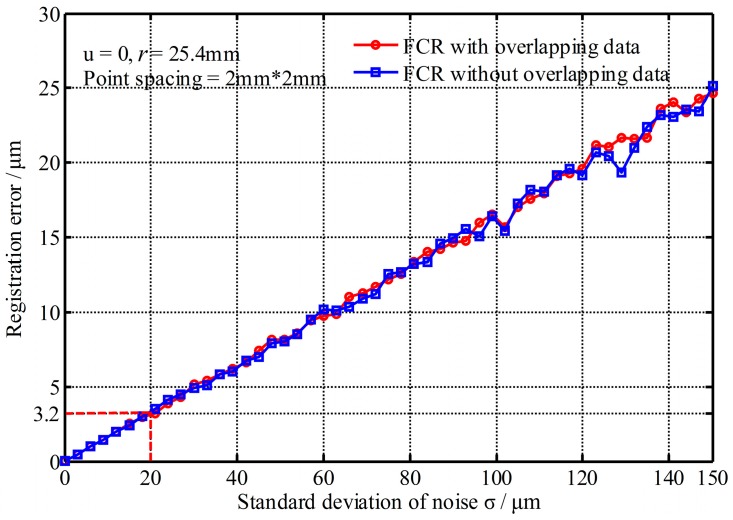
Registration errors changed with the standard deviation of noise.

Figure 7 shows the registration errors changed with the standard deviation of noise (mean error *u* = 0). It shows that the registration errors increase with the increase of the standard deviation of noise and the registration errors with and without overlapping data are still the same. However, the change rate is very small. Even the standard deviation reaches 150 μm, the registration error is only about 25 μm. Thus, the FCR method is not sensitive to the standard deviation of noise.

(2) Impact of the mean error

**Figure 8:**
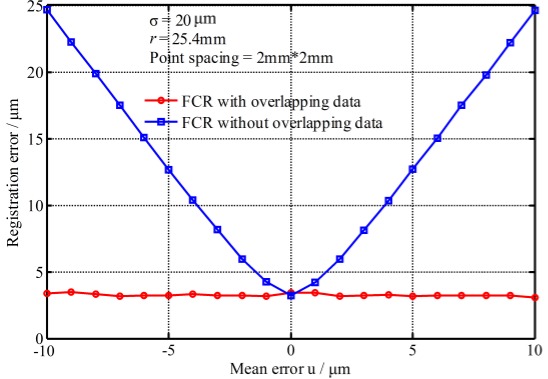
Registration errors changed with the mean error.

The mean error causes the fitting radius to have bias with the actual radius. Figure 8 shows the registration errors changed with the mean error. It shows that the registration errors with overlapping data almost do not change with the increase of the mean error, but the registration errors with non-overlapping data increase greatly with the increase of the mean error (absolute value). The reason is because of the radius constraint—namely, the radius bias—results in the registered point clouds deviating from their theoretical positions. However, for the two point clouds with complete overlap, their deviation positions are the same, and that counteracts the registration errors. So the mean error has great impact on the registration precision and it must be reduced or eliminated before registration. Fortunately the mean error of measurement represents a systematic error and usually can be eliminated or compensated in advance.

#### 4.1.4. Impact of the Overlap Ratio

**Figure 9:**
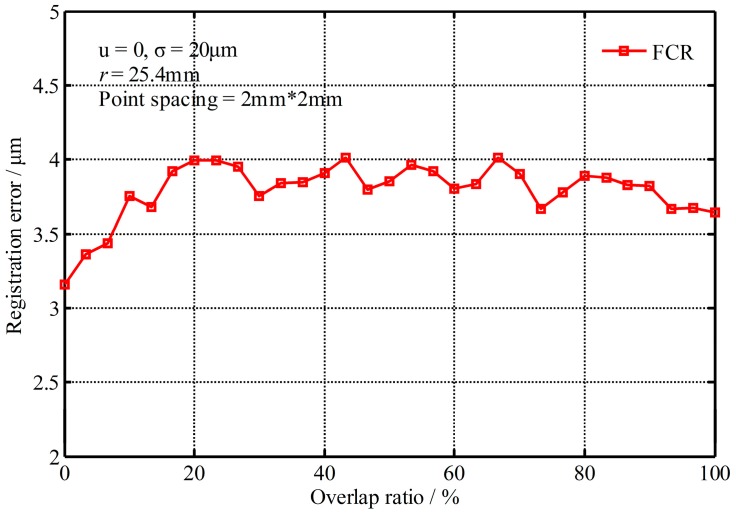
Registration errors changed with the overlapping ratio.

Figure 9 shows the registration errors changed with the overlapping ratio, where the overlap ratio represents the rate of overlapping areas to the registered points. From Figure 9, it is indicated that the registration errors change little (less than 1 μm) no matter how much the overlap ratio is. So the FCR method is not sensitive to the overlapping ratio, and it also verifies that the FCR method could overcome the problem of registration with non-overlapping data.

From the above analysis, it is demonstrated that the registration results with non-overlapping data are consistent with the results with overlapping data except for the mean error. Therefore, the FCR method is an effective method to register point clouds with non-overlapping data.

### 4.2. Comparison of Registration Precision with Typical Methods

To validate the precision of the FCR, SCR, and ICP methods are also applied to registering the simulation data and real experimental data, respectively. The simulation data with overlapping or non-overlapping data is generated with the same condition in Section 4.1. Simulations are repeated 500 times with different extraction points and re-generating random noise. The statistical results are listed in Table 1, where registration errors are obtained by Equation (8). From Table 1, the FCR method has the best registration precision, whose mean error is 3.3 μm and standard deviation of noise is 1.7 μm in both cases of containing overlapping data and non-overlapping data; SCR’s mean error is only about 11.4 μm and standard deviation of noise is 6.9 μm in those cases; while the mean error of ICP is 22.4 μm and the standard deviation of noise is 11.1 μm with the overlapping data, but it fails to register non-overlapping data.

In the real experiments with the measurement system [24,25], a standard ball with radius of 25.4 mm and a plate of 110 mm × 110 mm with three sphere targets are measured from multiple views to form overlapping data and non-overlapping data, respectively. The spherical shape error is less than 5 μm, and the flatness of the plate is higher than 5 μm. Only three sphere targets are applied to registration, while the ball and plate are just used as the evaluation object. The fitting residuals of the ball and plate are used as the registration errors, where the bias between the fitting radius and real radius value is calculated as the mean error for the ball. The statistical results of the registration errors are shown in Table 2. The registration precision is worse than the simulations, which results from the inaccurate experiment data, but Table 2 still shows that the FCR method has the highest precision, while ICP is the worst, which is similar to the simulation results.

Generally, the ICP algorithm is a high precision registration method, but the corresponding point pairs from the two extracted point clouds are not accurate enough, which is easy to converge to a local optimal solution and results in a wrong result. The noise causes errors in the fitting sphere centers, which makes the inaccurate alignment of the two point clouds with the SCR method. The FCR method further optimizes the transformation matrices with the radius constraint after obtaining a good initial value by the SCR method, and it gets more accurate corresponding point pairs from the constructed virtual surface instead of the extracted point cloud, which is a key factor in getting an optimal convergence result. Therefore, the FCR method achieves the best precision.

In addition, with 3400 simulating points per time, the running time of 500 times is also listed in the second column of Table 1. The results show that the registration speed of FCR is similar to ICP algorithm, but it is much slower than SCR.

### 4.3. Real Blade Registration

**Figure 10:**
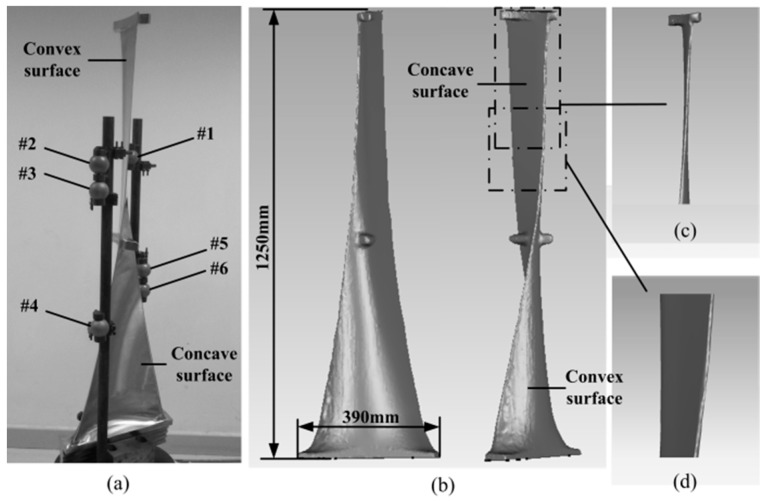
A large turbine blade for registration, (**a**) turbine blade with six sphere targets; (**b**) the completed profile of blade; (**c**) blade top section; and (**d**) transition segment between the upper and the middle sections.

A 1.25 m long turbine blade is measured from multi-view, and six sphere targets of *r* ≈ 25.4 mm (spherical shape error of those six sphere targets is less than 5 μm) are arranged nearby and fixed rigidly with the blade for registration, as shown in Figure 10a,b. The top section of turbine blade is an approximate sheet, which is difficult to obtain the overlapping data between the concave and convex surface, as shown in Figure 10c. The transition segment between the upper and the middle sections likes an approximate plane, which is hard to be registered because its curvature changes little or even unchanged, as shown in Figure 10d. Moreover, the concave and convex surfaces of the bottom only have few overlaps, which would decrease the registration precision. Obviously, it is hard to accurately register the point clouds of a blade by the existing methods.

**Figure 11:**
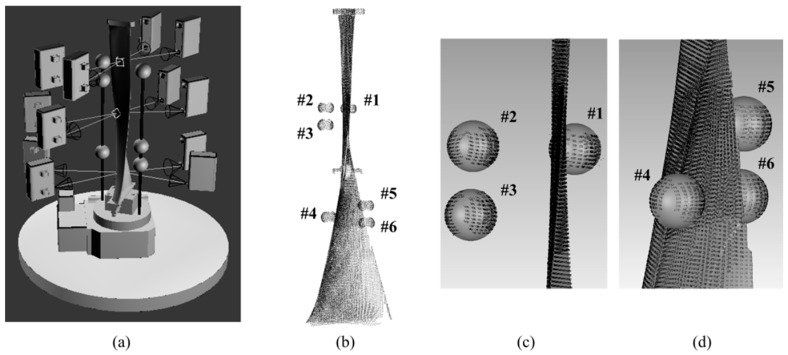
Registration of a turbine blade, (**a**) multiple measurement perspectives for the blade; (**b**) point cloud of blade after registration; (**c**) registration result of the upper segment; and (**d**) registration result of the bottom segment.

The blade is measured from top, middle, and bottom three segments, and each segment is measured from four perspectives, as shown in Figure 11a. Therefore, a total of 12 view fields need to be registered. With the FCR method, the upper three of the six sphere targets are applied to registration of the concave and convex surface of blade top section and registration of the upper and middle segments of blade; while the lower three targets are applied to registration of the blade bottom point clouds. The complete point cloud after registration is shown in Figure 11b; the registration results of the top section and transition segment are shown in Figure 11c, and the registration result of the bottom section is shown in Figure 11d. Figure 11 indicates that the point clouds are registered well.

**Figure 12:**
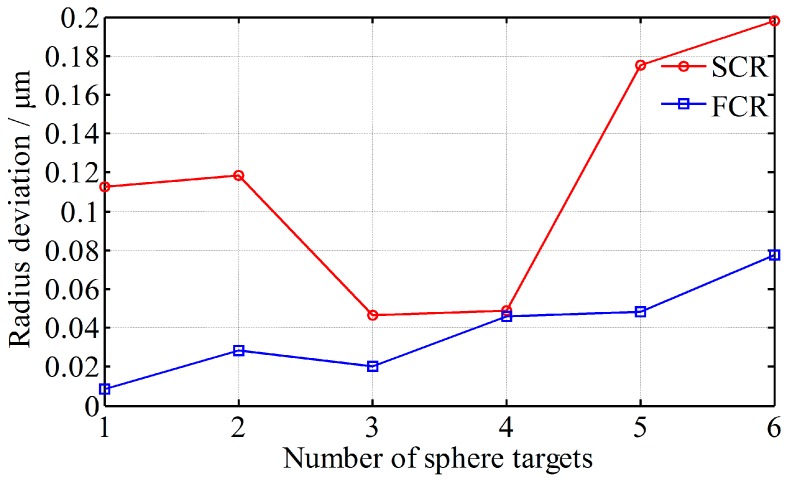
Radius deviation of the six sphere targets with FCR and SCR, respectively.

To evaluate the FCR’s registration precision, the measuring points of the six sphere targets are fitted to spheres, and the radius deviations between the fitting radii and actual radii are calculated and shown in Figure 12, where SCR is also adopted for comparison. The mean radius bias of FCR is about 0.0380 mm, and the mean bias of SCR is about 0.1166 mm. It indicates that the registration precision of FCR is much better than SCR.

Overall, the FCR method solves the problems of imprecise registration with non-overlapping areas or curvature invariant surface. Theoretically, its precision is not limited by the measured object size, and by only adding some feature targets, it is convenient to achieve a high-precision registration.

## 5. Conclusions

The paper proposes a sphere Feature Constraint Registration method (FCR), which is adapted for high-precision registration of the multi-view point clouds with non-overlapping areas or curvature invariant surface. The proposed method introduces some known sphere features to construct virtual overlapping areas for registration with non-overlapping areas point clouds, and utilizes the feature constraint to reduce the impact of the noise in point clouds and prevent the solution from falling into a local optimum. Even for the point clouds with overlapping areas, the proposed method is more accurate, since the corresponding points are selected from a continuous and ideal surface instead of the actual discrete measured points. The transformation parameters are solved by the optimization method with weight functions. In that case, the impact of large noise is reduced, and the registration stability is improved. Simulation and experiment results indicate that the proposed method solves the issues of impossible or low precision registration effectively. Moreover, the proposed method is less sensitive to the target size, point density, noise, and overlap ratio, so it is more accurate than the sphere center registration method and common ICP algorithm. Theoretically, not only sphere targets, but also others, such as cones, can be used as the feature targets in registration.

## Figures and Tables

**Table 1:** Registration results by three registration methods with simulation data.

Method	Running Time	Registration Error with Overlapping Data			Registration Error with Non-Overlapping Data		
		Mean	RMS	Max	Mean	RMS	Max
SCR	3 s	10.1 μm	6.7 μm	53.4 μm	11.4 μm	6.9 μm	60.2 μm
ICP	104 s	22.4 μm	11.1 μm	62.1 μm	/	/	/
FCR	96 s	3.3 μm	1.7 μm	11.6 μm	3.2 μm	1.7 μm	11.5 μm

**Table 2:** Registration results by three registration methods with real experimental data.

Object	Method	Registration Error with Overlapping Data			Registration Error with Non-Overlapping Data		
		Mean	RMS	Max	Mean	RMS	Max
Ball	SCR	0.036 mm	0.028 mm	0.144 mm	0.048 mm	0.033 mm	0.112 mm
	ICP	0.260 mm	0.130 mm	0.433 mm	/	/	/
	FCR	0.012 mm	0.023 mm	0.107 mm	0.009 mm	0.028 mm	0.119 mm
Plate	SCR	0	0.041 mm	0.121 mm	/	/	/
	ICP	0	0.104 mm	0.224 mm	/	/	/
	FCR	0	0.020 mm	0.069 mm	/	/	/

## Abbreviations

3D	Three-Dimensional
ICP	Iterative Closest Point
SCR	Sphere Center Registration
FCR	Feature Constraints Registration
CMM	Coordinates Measuring Machine
